# Is resistance ever free? Weighing the costs of altered strigolactone biosynthesis in sorghum

**DOI:** 10.1093/plphys/kiag692

**Published:** 2026-09-17

**Authors:** Neeta Lohani, Rita A Sharma

**Affiliations:** Assistant Features Editor, Plant Physiology, American Society of Plant Biologists; Department of Biotechnology, Thapar Institute of Engineering and Technology, Patiala, Punjab 147004, India; BRIC-National Agri-Food and Biomanufacturing Institute (NABI), Mohali, Punjab 140306, India

Strigolactones were first identified as germination stimulants for the parasitic plant *Striga* spp. (Cook et al. 1966), establishing a direct link between these carotenoid-derived hormones and parasite infestation. *Striga hermonthica* is a major constraint to cereal production and food security in tropical Africa, where it parasitizes several cereal crops, including sorghum, maize, and rice, causing billions of dollars of crop damage each year (Spallek et al. 2013). The susceptibility of sorghum, a globally important C4 cereal, is closely linked to its root-exuded strigolactones, and natural variation in these compounds.

Two enzymes, among several in the pathway, determine the strigolactone profile of sorghum root exudate. *CAROTENOID CLEAVAGE DIOXYGENASE 8* (*SbCCD8b*) acts early in the pathway and governs total strigolactone production, whereas *LOW GERMINATION STIMULANT 1* (*LGS1*), a sulfotransferase acting downstream, determines which strigolactone predominates. Genotypes with functional *LGS1* exude mainly 5-deoxystrigol (5DS), a potent germination stimulant, whereas loss-of-function genotypes exude predominantly orobanchol and stimulate substantially less germination (Gobena et al. 2017). These loss-of-function alleles are therefore attractive targets for breeding *Striga* resistance into farmer-preferred varieties (Ejeta 2007). They are chromosomal deletions, and several remove neighboring genes in addition to *LGS1*, so resistance at this locus reflects the loss of a gene block rather than of a single gene. However, their effects are not confined to the parasite interaction. Strigolactones also function as endogenous hormones regulating development and physiology, and rhizosphere signals recruiting arbuscular mycorrhizal fungi (AMF), which *Striga* exploits to locate a host, raising the question if altering strigolactone biosynthesis to confer parasite resistance compromises these functions.

In this issue of *Plant Physiology*, McLaughlin et al. (2026) address this question by examining the consequences of altering strigolactone biosynthesis in 2 sorghum inbred backgrounds that differ naturally at *LGS1*. Macia carries a functional *LGS1* allele, whereas RTx430, a widely used breeding line, carries the *lgs1-2* deletion. This deletion spans 3 genes: *LGS1* (Sobic.005G213600), *Sb3500* (Sobic.005G213500), and Sobic.005G213400. *Sb3500* encodes a 2-oxoglutarate-dependent dioxygenase that increases 5DS production when co-expressed with *LGS1* (Yoda et al. 2023).

Three engineered lines allowed the authors to evaluate strigolactone quantity and composition across genetic backgrounds expected to differ in these traits. RTx430 *ccd8b* is a CRISPR-Cas9 deletion of *SbCCD8b* (Hao et al. 2023); Macia *lgs1-d* is a CRISPR-Cas9 deletion of *LGS1* (Bellis et al. 2020), backcrossed to Macia for this study; and RTx430 *LGS1* is a gain-of-function insertion carrying *LGS1* alone, without the neighboring genes present in the *lgs1-2* deletion. These lines were grown under high phosphate, low phosphate, and low phosphate with mycorrhizal inoculation. The authors measured photosynthetic and stomatal traits, root architecture and anatomy, and mycorrhizal colonization at 21 and 42 days. Parasite response was measured by exposing *Striga* seeds to sorghum root exudates (Fig. 1a).

**Figure 1:**
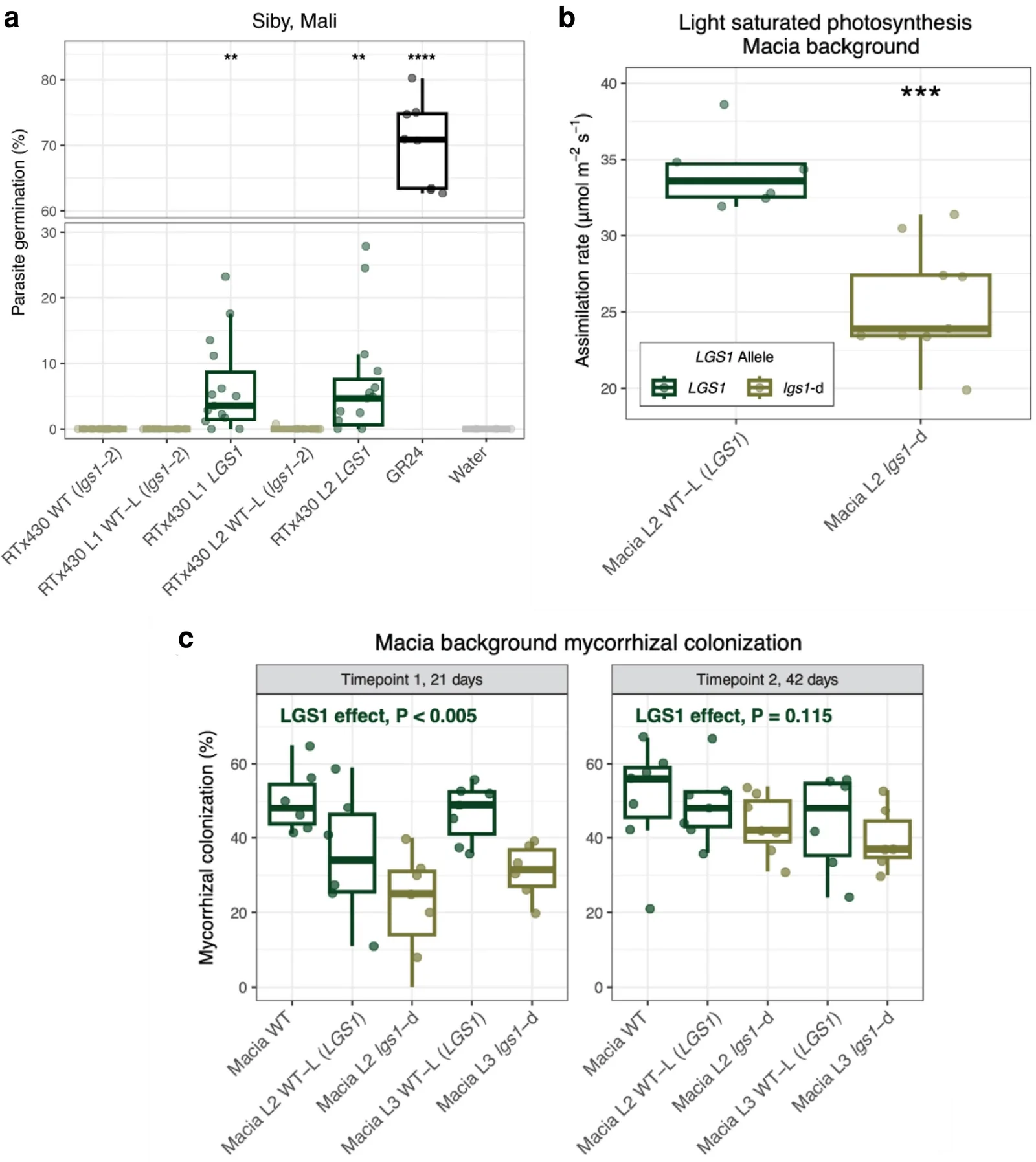
Restoring *LGS1* in the RTx430 background restores the germination cue for *Striga hermonthica*, whereas deleting *LGS1* in Macia reduces photosynthesis and delays mycorrhizal colonization. a) *Striga hermonthica* germination (Siby, Mali ecotype) in response to root exudate from RTx430 wild-type, null segregants, and *LGS1* insertion mutants, with GR24 and water as controls. b) Light-saturated assimilation rate of Macia *LGS1* deletion mutants (*lgs1-d*) and null segregants. c) Roots colonized by *Rhizophagus intraradices* at 21 and 42 days in Macia genotypes differing in *LGS1* functionality. Dark green marks functional LGS1 in all panels. Pale green in a) marks the lgs1-2 deletion background (RTx430 wild type and null segregants); olive in b) and c) marks the lgs1-d deletion. Adapted from McLaughlin et al. (2026).

The strigolactone-deficient line RTx430 *ccd8b*, which exudes almost no strigolactones and does not stimulate *Striga* germination (Hao et al. 2023), established the upper bound of the cost. In all 3 treatments, including the no-nutrient limitation, these plants were significantly shorter, accumulated less shoot and root biomass, and showed reduced light-saturated carbon assimilation. Root systems were smaller with reduced aerenchyma (the air spaces in the root cortex). Mycorrhizal colonization was poor at both 21 and 42 days, and inoculation conferred 20% less shoot-weight gain than in wild-type plants. This genotype represents an experimental extreme rather than natural variation, but it defines what a sorghum plant loses when the strigolactone pathway is disabled.

Deleting *LGS1* alone in Macia produced milder but consistent costs. Light-saturated photosynthesis declined by approximately 25% (Fig. 1b), as did the maximum rate of Rubisco carboxylation. Chlorophyll content and photosystem protein abundance were unchanged. Lower Rubisco activity or activation state, rather than structural change in the photosynthetic apparatus, however, this still needs validation. Photosynthesis genes are downregulated in Macia lgs1-d shoots (Bellis et al. 2020). Here, sequences flanking those differentially expressed genes, which span photosynthesis and other functional categories, were enriched for binding motifs of transcription factors linked to development and stress responses, pointing to a broader regulatory effect. Mycorrhizal colonization was 48% lower at 21 days but had narrowed to a nonsignificant 15% difference by 42 days (Fig. 1c). Loss of *SbCCD8b* therefore compromises the symbiosis, whereas loss of *LGS1* delays it.

The reciprocal experiment proved most informative. Reintroducing *LGS1* into RTx430 separated the gene's effect on *Striga* germination from its influence on host physiology. Root exudate from the insertion lines stimulated substantial germination, whereas exudate from RTx430 did not (Fig. 1a). Photosynthesis and mycorrhizal colonization, however, matched those of RTx430. Epistasis with the neighboring genes may account for this dissociation, although the RTx430 and Macia backgrounds differ in far more than this locus. Because the *lgs1-2* deletion also removes *Sb3500*, the insertion line carries *LGS1* without its enzymatic partner and is predicted to produce roughly equal amounts of 5DS and 4-deoxyorobanchol rather than the 5DS-dominant blend of intact Macia. The phenotypic effect of *LGS1,* may therefore, depend on which neighboring alleles sit beside it. Allele combinations in landraces are also consistent with this interpretation. Out of 704 accessions, 668 retain both *LGS1* and *Sb3500*, and only one carries the *LGS1-*without*-Sb3500* combination that the insertion line reproduces. That rarity fits selection, maintaining the module, and may also indicate fitness costs these assays did not capture.

Additionally, the predicted strigolactone profiles also rest on published enzyme activities rather than direct measurement, so restoring *Sb3500* alongside *LGS1* and quantifying the resulting exudate remains the direct test of this model. RTx430 carries other natural mutations besides the *lgs1-2* deletion, so its background effect cannot be assigned to *Sb3500* alone, and noncanonical strigolactones may carry much of the endogenous signaling load (Ito et al. 2022), limiting what canonical-pathway mutants can reveal. Testing the epistasis directly will need backgrounds that segregate for presence or absence of both LGS1 and Sb3500.

Therefore, the cost of resistance at this locus appears conditional, and for breeders, the findings of this study are cautionary rather than discouraging. An allele that reduces photosynthesis in one background need not do so in another, so the recipient variety matters as much as the allele chosen. Furthermore, greenhouse experiments cannot reproduce field conditions, and as the authors note, whatever cost a deletion carries may still be preferable to the crop lost to *Striga*. Therefore, the question for sorghum improvement is no longer whether *LGS1* can be altered to confer resistance. It is which combinations of alleles at this locus confer resistance while preserving the developmental, photosynthetic, and symbiotic roles of strigolactones.

## Recent research articles in Plant Physiology:

- **Suga et al. (2026)** identified HINC55, a quinone signaling inhibitor that blocks prehaustorium formation in *Striga hermonthica* and *Phtheirospermum japonicum* and, in *Striga*, suppresses induction by quinones and phenolics but not by cytokinins, providing a chemical tool for dissecting host-parasite communication (https://doi.org/10.1093/plphys/kiaf686)
- **Singh et al. (2026)** phenotyped the Sorghum Association Panel (n = 397) for leaf anatomical and physiological traits and identified a genomic hotspot on chromosome 1 whose haplotypes influence stomatal density, assimilation rate, stomatal conductance, and intrinsic water-use efficiency, the same carbon-water balance perturbed in strigolactone mutants (https://doi.org/10.1093/plphys/kiag064)

## Data Availability

No new data were generated or analyzed in support of this article.

## References

[kiag692-B1] Bellis ES et al 2020. Genomics of sorghum local adaptation to a parasitic plant. Proc Natl Acad Sci U S A. 117:4243–4251. 10.1073/pnas.1908707117. PMC7049153 32047036

[kiag692-B2] Cook C, Whichard LP, Turner B, Wall ME, Egley GH. 1966. Germination of witchweed (Striga lutea Lour.): isolation and properties of a potent stimulant. Science. 154:1189–1190. 10.1126/science.154.3753.1189. 17780042

[kiag692-B3] Ejeta G . 2007. Breeding for *Striga* resistance in Sorghum: exploitation of an intricate host–parasite biology. Crop Sci. 47:S-216–S-227. 10.2135/cropsci2007.04.0011IPBS.

[kiag692-B4] Gobena D et al 2017. Mutation in sorghum LOW GERMINATION STIMULANT 1 alters strigolactones and causes Striga resistance. Proc Natl Acad Sci U S A. 114:4471–4476. 10.1073/pnas.1618965114. PMC5410831 28396420

[kiag692-B5] Hao J et al 2023. CRISPR/Cas9-mediated mutagenesis of carotenoid cleavage dioxygenase (CCD) genes in sorghum alters strigolactone biosynthesis and plant biotic interactions. Phytobiomes J. 7:339–351. 10.1094/PBIOMES-08-22-0053-R.

[kiag692-B6] Ito S et al 2022. Canonical strigolactones are not the major determinant of tillering but important rhizospheric signals in rice. Sci Adv. 8:eadd1278. 10.1126/sciadv.add1278. PMC9629705 36322663

[kiag692-B7] McLaughlin CM et al 2026. Strigolactone effects on Sorghum bicolor ecophysiology and symbioses. Plant Physiol. 10.1093/plphys/kiag549. 42497307

[kiag692-B8] Singh A, Newton L, Thompson AM. 2026. Genome-wide association studies and modeling of stomatal gas conductance reveal genetic control of water-use efficiency in sorghum. Plant Physiol. 200:kiag064. 10.1093/plphys/kiag064. PMC13016993 41698033

[kiag692-B9] Spallek T, Mutuku M, Shirasu K. 2013. The genus Striga: a witch profile. Mol Plant Pathol. 14:861–869. 10.1111/mpp.12058. PMC6638688 23841683

[kiag692-B10] Suga S et al 2026. A quinone signaling inhibitor enables functional dissection of haustorium-inducing factors in Orobanchaceae parasitic plants. Plant Physiol. 200:kiaf686. 10.1093/plphys/kiaf686. 41604449

[kiag692-B11] Yoda A et al 2023. A stereoselective strigolactone biosynthesis catalyzed by a 2-oxoglutarate-dependent dioxygenase in sorghum. Plant Cell Physiol. 64:1034–1045. 10.1093/pcp/pcad060. PMC10504574 37307421

